# Supplementary material for: Adherence to COVID-19 policy measures: Behavioral insights from The Netherlands and Belgium
Source: PLoS One. 2021 May 28;16(5):e0250302. doi: 10.1371/journal.pone.0250302 (PMC8162620; doi:10.1371/journal.pone.0250302)
Supplement: Attachment — Submitted filename: Response to Reviewers.docx [file pone.0250302.s003.docx]

1. Please ensure that your manuscript meets PLOS ONE's style requirements, including those for file naming. The PLOS ONE style templates can be found at

<https://journals.plos.org/plosone/s/file?id=wjVg/PLOSOne_formatting_sample_main_body.pdf> and<https://journals.plos.org/plosone/s/file?id=ba62/PLOSOne_formatting_sample_title_authors_affiliations.pdf>

We edited the manuscript and title page according to the PLOS ONE style templates.

2. We suggest you thoroughly copyedit your manuscript for language usage, spelling, and grammar. If you do not know anyone who can help you do this, you may wish to consider employing a professional scientific editing service.

We had our manuscript copyedited at the University of Vermont.

3. We note that you have indicated that data from this study are available upon request. PLOS only allows data to be available upon request if there are legal or ethical restrictions on sharing data publicly. For information on unacceptable data access restrictions, please see <http://journals.plos.org/plosone/s/data-availability#loc-unacceptable-data-access-restrictions>.

We have now shared our data from this study to a public repository as well as with a supporting information file with this submission. Please update the Data Availability statement on our behalf. The public repository can be found here: <https://github.com/ElinevandenBroek/Covid-19-NL-BE-data>

4. Please include a copy of Tables 6 and 7 which you refer to in your text on page 20.

This was a typo, this paragraph refers to table 4. We edited the text.

5. Please include captions for your Supporting Information files at the end of your manuscript, and update any in-text citations to match accordingly. Please see our Supporting Information guidelines for more information: [http://journals.plos.org/plosone/s/supporting-information](about:blank).

We do not have supporting information files in our revision, other than the data from this study which will not be included in the in-text citations.

Reviewer #1: The study entitled “Adherence to COVID-19 Policy Measures: Behavioral Insights from The Netherlands and Belgium” investigated the individual variation in adhering the behavioral recommendations made by the government during the COVID-19 pandemic. The roles of motivations, beliefs and pre-pandemic behavior in the policy response were evaluated. The findings indicate that the citizens in Netherlands and those in Belgium had different attitudes and behaviors reacting to the COVID-19. Therefore, the authors suggested that it is not recommended to adopt “one-size-fits-all” policy strategies to mitigate the spread of COVID-19. The manuscript tackles an important and timely research question with the following strengths: (1) a large sample size; (2) cross-country comparison; and (3) rigorous statistical methods. However, several issues should be remediated before I recommend publication. Please see my specific comments below.

1. The Abstract is not in a good shape. I think that Plos One allows much more words than the current presented Abstract. The authors are thus needed to provide more details and findings (with statistical values) in the Abstract. I would also prefer to see a structured Abstract as this is clear for readers to have a full picture in the beginning of reading this paper.

Thank you very much for your suggestion. We have rewritten the abstract in a structured form and added more details and findings on pages 1 and 2.

2. The authors do not review the literature thoroughly. Specifically, many papers on policy, behaviors, and associated factors during COVID-19 pandemic have been published. Therefore, the authors should take references from them. Below are some of my suggestions for the authors to use and strengthen their literature review.

We appreciate the feedback and have added a more thorough review of the literature, including the reviewer’s suggestions below.

Olatunji OS, Ayandele O, Ashirudeen D, Olaniru OS. “Infodemic” in a pandemic: COVID-19 conspiracy theories in an african country. Soc Health Behav 2020;3:152-7

Lin MW, Cheng Y. Policy actions to alleviate psychosocial impacts of COVID-19 pandemic: Experiences from Taiwan. Soc Health Behav 2020;3:72-3

Rieger MO. To wear or not to wear? Factors influencing wearing face masks in Germany during the COVID-19 pandemic. Soc Health Behav 2020;3:50-4

Chang KC, Strong C, Pakpour AH, Griffiths MD, Lin CY. Factors related to preventive COVID-19 infection behaviors among people with mental illness. J Formos Med Assoc. 2020;119(12):1772-1780. doi:10.1016/j.jfma.2020.07.032

Pramukti I, Strong C, Sitthimongkol Y, Setiawan A, Pandin MGR, Yen CF, Lin CY, Griffiths MD, Ko NY. Anxiety and Suicidal Thoughts During the COVID-19 Pandemic: Cross-Country Comparative Study Among Indonesian, Taiwanese, and Thai University Students. J Med Internet Res 2020;22(12):e24487

3. I cannot understand why the authors reported t-values for categorical data on Table 1. Also, the meaning of the asterisks is not provided for Table 1.

We have added caption for Table 1, explaining that the asterisks in error and deleted those from the table.

4. The meaning of social 10+ is unclear for Tables 2 to 4.

This means social gatherings of 10 or more people. We added this caption below the tables 2 tot 4.

5. The study should have a limitation section to remind the readers, especially I found that the study did not use standardized instruments for assessing all the behaviors (e.g., Chang et al. have validated behavioral instruments during COVID-19). Therefore, it is unclear whether the measures used in the present study are robust.

Thank you for this suggestion. We have added a Limitation section after the Discussion section on page 24. We have addressed this issue explaining why we used these methods not using standardized instruments, citing Change et al. and added text.

Chang KC, Hou WL, Pakpour AH, Lin CY, Griffiths MD. Psychometric Testing of Three COVID-19-Related Scales Among People with Mental Illness [published online ahead of print, 2020 Jul 11]. Int J Ment Health Addict. 2020;1-13. doi:10.1007/s11469-020-00361-6
